# Clinical decision-making: midwifery students' recognition of, and response to, post partum haemorrhage in the simulation environment

**DOI:** 10.1186/1471-2393-12-19

**Published:** 2012-03-23

**Authors:** Julie Scholes, Ruth Endacott, MaryAnne Biro, Bree Bulle, Simon Cooper, Maureen Miles, Carole Gilmour, Penny Buykx, Leigh Kinsman, Rosemarie Boland, Jan Jones, Fawzia Zaidi

**Affiliations:** 1 Centre for Nursing and Midwifery Research, University of Brighton, Mayfield House, Village Way, Brighton, UK; 2 Faculty of Health and Social Work, University of Plymouth, Drake Circus, Plymouth, UK; 3 School of Nursing & Midwifery, Monash University, Clayton Campus, Victoria, Australia; 4 The Royal Women's Hospital, Melbourne, Victoria, Australia; 5 School of Nursing (Berwick), Monash University, Berwick, Victoria, Australia; 6 School of Nursing and Midwifery, Monash University, Peninsula Campus, Frankston, Victoria, Australia; 7 School of Rural Health, Monash University, Bendigo, Victoria, Australia; 8 Nurse Educator: NETS (Victoria), PhD Scholar, University of Melbourne, Melbourne, Australia; 9 School of Nursing and Midwifery, Monash University, Gippsland Campus, Churchill, Victoria, Australia

## Abstract

**Background:**

This paper reports the findings of a study of how midwifery students responded to a simulated post partum haemorrhage (PPH). Internationally, 25% of maternal deaths are attributed to severe haemorrhage. Although this figure is far higher in developing countries, the risk to maternal wellbeing and child health problem means that all midwives need to remain vigilant and respond appropriately to early signs of maternal deterioration.

**Methods:**

Simulation using a patient actress enabled the research team to investigate the way in which 35 midwifery students made decisions in a dynamic high fidelity PPH scenario. The actress wore a birthing suit that simulated blood loss and a flaccid uterus on palpation. The scenario provided low levels of uncertainty and high levels of relevant information. The student's response to the scenario was videoed. Immediately after, they were invited to review the video, reflect on their performance and give a commentary as to what affected their decisions. The data were analysed using Dimensional Analysis.

**Results:**

The students' clinical management of the situation varied considerably. Students struggled to prioritise their actions where more than one response was required to a clinical cue and did not necessarily use mnemonics as heuristic devices to guide their actions. Driven by a response to single cues they also showed a reluctance to formulate a diagnosis based on inductive and deductive reasoning cycles. This meant they did not necessarily introduce new hypothetical ideas against which they might refute or confirm a diagnosis and thereby eliminate fixation error.

**Conclusions:**

The students response demonstrated that a number of clinical skills require updating on a regular basis including: fundal massage technique, the use of emergency standing order drugs, communication and delegation of tasks to others in an emergency and working independently until help arrives. Heuristic devices helped the students to evaluate their interventions to illuminate what else could be done whilst they awaited the emergency team. They did not necessarily serve to prompt the students' or help them plan care prospectively. The limitations of the study are critically explored along with the pedagogic implications for initial training and continuing professional development.

## Background

This paper reports the findings of a study undertaken to examine student midwives' response to obstetric emergencies. This work is part of a portfolio of research examining the response of student nurses, midwives and latterly qualified nurses, to rapid patient deterioration in a simulation setting [1,2]. The paper will focus on how midwifery students responded to a simulated post partum haemorrhage (PPH), how they made decisions to inform their response and critically explore the methods by which these data were gathered and analysed.

The World Health Organisation estimated that severe bleeding and haemorrhage accounts for 25% of maternal deaths [3] with post partum haemorrhage the leading cause of maternal death and morbidity [4]. Although PPH as the cause of maternal death is 100 times higher in developing countries, it is a significant risk to maternal wellbeing and child health in developed countries. For example, the current rate of PPH in Victoria, Australia is 11.9% [5]. In the UK, the incidence of PPH is on the decline [6] with the number of maternal deaths from PPH halved to 5 in the reporting period 2006-2008 [7]. This has been attributed to prophylactic oxytocic administration, and the swift and skilled multidisciplinary response to a haemorrhage once it has been recognised [7]. This means that midwives and obstetricians need to be vigilant and highly skilled in identifying and managing very sick women and making timely and appropriate interventions to reduce preventable harm [8].

Suboptimal clinical care contributed to 67% of maternal deaths in the UK 2003-05 [9] but three out of the five deaths reported in the period 2006-8 were due to a lack of routine observation and response to rapidly changing haemodynamic measurement [7]. Substandard care (SSC) is attributed to 70% of direct maternal deaths which include PPH as a cause [8]. Similar recommendations have been stated in The Confidential Inquiry into Maternal and Child Health (CEMACH) report [10] and latterly in the CMACE 2011 report [7] to address SSC. They include: improvements in communication notably reporting concerns 'upwards' to more senior colleagues; improvements in senior support; the use of early warning scores to help identify sick women; general improvements in the clinical knowledge and skills of midwives and doctors to help manage emergency situations that include basic and advanced life support skills; better management of higher risk women and reviewing and learning from serious incidents or untoward incident reporting [8].

### Simulation: enhancing midwives' emergency response to the critically ill women

Simulation has been used to support training in high stress situations that would be unsafe to rehearse in clinical practice [11]. It offers the opportunity for learning from error without causing harm to the patient [12], competence acquisition [13], and the development of clinical reasoning skills [14]. The experience can be enhanced if the simulation is as close to clinical reality as possible, known as high fidelity [15]. Simulation has many advantages in teaching and learning about crisis or emergency situations [16]. However, the students' willingness to engage authentically in the scenario is essential to maximize the learning potential of simulation [17,18]. Expert facilitated debriefing is essential to maximize the benefits of learning from such an experience and also to handle the emotions associated with this type of learning [19].

Simulation environments are rapidly expanding and this provides opportunity to research decision-making and skill acquisition in high fidelity complex scenarios [11]. The addition of actors linked to computerized manikins enhances the fidelity of the situation [19]. Cioffi [20] argues the use of simulation can enhance clinical decision making of midwifery students. Therefore when using simulation to assess clinical decision making participation in the research can of itself act as an educative opportunity [21].

Thinking aloud techniques enable a fuller exploration of the decision-making process in simulation scenarios capturing what is held in the short-term memory [20]. Video-cued reflection is an alternative approach that can capture critical analysis of the individual's response to an emergency situation [17]. It enables the researcher to gain insight into the participants' tacit understanding [22]. Guided facilitation through the process is important to ensure the participant is fully engaged in the review and to enable them to provide an account that is more than an introspective analysis of their actions [17].

### Decision making in midwifery

Midwives would ideally consider their approach to decision making to be founded on a partnership model with the mother. In critical illness, the mother's compromised capacity to share decision-making shifts this partnership model [23] to one where the midwife has to assume temporary control. A midwife has to draw upon irregularly rehearsed skills to respond effectively to the situation, use more systematic approaches to decision-making and defer to expert medical intervention [23]. Institutional and organizational factors will prevail in times of emergency [24]. The student midwife needs to learn how to respond to rapid physical deterioration under pressure.

Theoretical decision-making can be examined through three perspectives: prescriptive; normative and descriptive [25]. Prescriptive theory assumes error and examines ways in which for example, clinical decisions systems can facilitate timely and accurate decisions. Normative theory focuses on rational decision-making and how to optimise authority, reason and conscience, acknowledging that these decisions are influenced by social, organisational and contextual factors. The final typology, descriptive decision theory, focuses upon the process of decision-making, the factors that influence a decision including complexity, affect, time and other reference points influencing a decision, including emotion. The approach examines the use of heuristic devices to assist memory recall and facilitate mental shortcuts to facilitate decision-making. However, it is these heuristic devices that can lead to initial fixation error, bias or contradiction. The work of Coiffi [20,26] has explored descriptive decision theory to explain midwifery decision-making and the importance of heuristic devices, whilst Rattray [27] offers prescriptive solutions (such as clinical decision-making systems) as a prompt to assist timely and accurate decisions in the use of fetal monitoring in low risk labouring women. In general, the greater the risk and likelihood of adverse outcomes, prescriptive solutions are advocated by an organisation or central policy. In the case of managing post partum haemorrhage, the policy set out in CEMACH and CMACE directed institutions to introduce early warning scores and protocols to determine the clinical response [7,10]. However, bureaucratic decision-making, driven by risk and litigation averse policies and procedures were found to be dominant in midwifery decision-making in their every day work, not just emergencies [27,28].

This study has taken a descriptive approach to explore how:

1) student midwives make decisions and respond to an obstetric emergency

2) we can enhance student midwives' decision-making and clinical skills in response to a simulated PPH.

## Methods

Students were exposed to instruction on managing maternal deterioration and response to obstetric emergency as part of their curriculum programme. Thirty five students were recruited to the research arm of the study. They agreed to participate in:

• Video recordings of their performance in the simulation environment

• Video-cued narrative reflection of their performance

• An evaluation of their experience

• A knowledge questionnaire

When the students entered the clinical laboratory, they were provided with minimal information about the mother's biographical details, an outline history of her labour and birth and current status, and reasons for presentation to the unit leaving them to seek out further information to inform their assessment.

The students interacted with an actress 'Lisa' who enacted rapid onset shock in response to PPH. She wore a birthing suit that simulated a poorly contracted uterus and blood loss. The actress performed to a script and manifest behaviour that simulated rapid physiological deterioration e.g. changing levels of consciousness and pain. This created ecological validity in the simulation [23] so the student could experience clinical thinking under stress and the researchers were able to examine decision making in dynamic high fidelity scenarios. Students were provided debriefing sessions following the experience.

The scenario had been constructed from an amalgam of maternal case studies. A panel of clinical experts assessed the scenarios to confirm content and face validity. The scenarios were constructed to last for 8 minutes. In the first four minutes the mother presented with subtle signs of deterioration, but the maternal condition deteriorated rapidly in the final 4 minutes. The clinical presenting symptoms were obvious (low levels of uncertainty and high levels of relevant information).

The student conducted the simulation individually. They were able to interact with a researcher who posed as a junior doctor. The junior doctor was 'unhelpful' and was there only to provide medical interventions on request, for example prescribing medication or undertaking medical procedures. Although stressful to the students, the focus of inquiry was on the students' capacity to recognise and respond to deterioration and make timely requests for assistance and drive upwards their calls for assistance to more senior staff. The delay in the arrival of the emergency response team was considered to be a realistic potential that enabled the research team to observe the full repertoire of the students' emergency skills.

After the scenario the students were invited to review their performance recorded on video and provide commentary on the decisions that prompted their actions. The student was in control of the initial analysis of their performance. The facilitator would pose reflective questions if the student was uncommunicative [29] e.g.

What did you notice?

Why did you ask that (piece of information from vignette)

What were you looking for?

What triggered you to [x y] action?

What are your concerns for the woman?

Each question was triggered by the action on the video. No judgement was made on performance, unless so weak it raised questions about fitness to practice. Remedial tutorials were provided if serious knowledge and performance deficits were identified. Of note, students were self critical, demonstrating insight into their capabilities and the limitations of their competence.

### Ethical considerations

The xxxx University Human Research Ethics Committee approved the study. Students were invited to participate through posters and invitations from the research team. Staff members who did not have a direct power relationship with the student on their course took consent. Emphasis was placed upon the debriefing components of the study and the potential for additional training if the student felt this was necessary. Additional consent to use video materials for conferences and teaching purposes was obtained after the debriefing session. Students were made aware that two (unknown) members of the research team, based in the UK, would undertake video analysis.

### Data analysis

Video data and interview materials were analysed using an adapted form of dimensional analysis [DA] [30]. Consistent with DA the description of the analytical process includes some initial findings. The video footage acted as the 'field' and was subject to repeated observation and analysis. Initially, key events, timings and activities were extracted from the observation. These data demonstrated the responses of the students to the simulation. These data were loaded into a grid to compare across cases the sequence of events leading up to actions. The grids for activities expanded as new events were identified and this caused a review of previous participants' footage. Thus data analysis did drive data collection but in this instance through further video review once incidents of analytical importance had been recognised and understood. Thus the grid expanded as more activities were included and conflated as analysis merged similar themes.

The analyses focused upon processes inherent to decision making i.e. activities were linked to the context, conditions, processes and consequences of actions. From these grids we were then able to aggregate examples of data into like groups to see the analytical possibility. Each time new grids were formed - they were headed by an analytical memo and ended with the theoretical memo determining where data might be further interrogated and compared (see sample of the analytical approach in Table 1).

**Table 1:** Data extraction grid: calling for assistance and the generation of different types of analytical memos.

Analytical memo 1) What type of assistance do students seek and at what time? What is the pattern of escalating calls for assistance			
	**Buzzer**	**Specified medical help**	**MET**

1	x		**✓ **6.11

2	x	Get the Dr 1.59	X

3	x	Get another person 3.25	X

4	**✓ **3.22	Get the dr 3.42	**✓ **5.57

5	**✓ **3.24		**✓ **8.02

6	**✓ **2.44		x

7	**✓ **5.09		CODE 6.34

8	**✓**5.45	Cal the DR 5.45	x

9		Cal the DR 3.41	**✓ **6.06

10			x

11			Code Blue 6.34

12		Interacts/instructs DR in room	x

13	**✓ **5.13	Dr to get Help & PPH kit 0.57	**✓ **6.59

14		Report findings to DR 3.50	Code blue 7.23

15			**✓ **7.05

16	**✓ **3.21		X

17	**✓ **3.25		x

18	**✓ **4.11		**✓ **4.55,6.53.7.08

19	**✓ **4.10		**✓ **7.01

20	**✓ **4.11		**✓ **7.22

21	**✓ **47 secs check several time on the way?		**✓ **5.18

22	**✓**6 44		**✓ **6.31

23	**✓ **5.53	DR Call the Reg	X

24		6.42 More help	X

25	**✓ **2.15 call help		X

26		Dr come & check 2.57	X

27		Working with jnr DR	X

28		Help asked of jnr DR	X

29	**✓ **506	Wants help 3.11	X

30		Working with jnr DR	X

31	**✓ **0.59		X

32	**✓ **4.52		X

33	x	x	X

34	**✓ **1.37		X

35	**✓ **2.07		**✓ **6.19

Analytical memos

• Align all those who make one call for assistance, against those who escalate calls for assistance (can you see patterns in date of data collection/students' course/campus on which data were collected? (form new comparative grids with these headings)

• Return to individual data: what happens before they call for help how much data have they assimilated before seeking assistance (from whom)?

• What triggers (clinical cues, interactions, activities) precede escalating calls for help

• Have the students labelled the emergency a PPH before they call for help? Does this affect who they call for help?

Field Memo: What is the difference in a Green Code, Code Blue, MET, emergency buzzer in the different clinical placements? Do they all trigger generic emergency response teams or an emergency obstetric team?

**Table 2:** Summary of the students' response (n = 35) to PPH scenario using key clinical management strategies

Fundal massage	Oxytocic administration		Assessing blood loss	Bladder care	Calling for help		Checking placenta
Xx (68%) of the students performed intermittent fundal massage xx (30%) performed continuous fundal massage	Correct oxytocic in timely manner 18 students (xx%) requested oxytocics before IV access, 14 students (40%) requested Syntocinon and 4 (11%) requested Ergometrine or Syntometrine (correct first line management for PPH)	Non first line oxytocic: 14 students (40%) requested Syntocinon before IV access	Blood loss: All 35 students (100%) visually checked blood loss in the PPH catheter scenario	Bladder: 10 students (28%)discussed or inserted an indwelling urinary	Local doctor: 11 students (31%) articulated a need for medical help	MET: 15 students checked escalate to a met call 15/35 (42%)	13 students status of placenta 13/35 (37%)

In the next phase of analysis we lined up reflective interview data with the grid to provide deeper understanding of why actions were taken. For example, participant 10 called for a Dr and more midwives at 3 minutes 41 seconds (prior to the onset of rapid deterioration), based on her assessment of the mother's blood loss and pain. She did not seek any help or further assistance after the maternal collapse. When questioned about her performance she replied:

'I took her vital signs to start with and then her loss looked fine to me at the start. I checked again and she was bleeding. I started to eliminate the causes of her bleeding, the tone of her uterus, her placenta was complete. She had a first-degree tear that was sutured [in the scenario she is told the mother had a first degree tear that did not require sutures]. The only thing was her thrombin, clotting factors, I tried to administer meds to help it [medications administered included: syntocinon and ergometrine. In the scenario the student had asked for bloods to be taken but did not specify thrombin]. It helped a little, but not really. I didn't know what other steps to take, I knew if it didn't stop she may have to go down to theatre.

• The student stated in her reflective interview that she was certain that the mother was experiencing a post partum haemorrhage although she was uncertain as to the cause of the continual bleeding. The students had been taught to respond to a PPH using the mnemonic, the 4 Ts (Tone, Tissue, Trauma, Thrombin) and each cause's associated clinical management: Uterine atonia is the commonest cause of PPH, so students had been taught to manage this in the first instance. The students' response to the PPH scenario will be presented under the following responses:

• Rubbing up the fundus

• Oxytocic administration

• Assessing Blood Loss

• Bladder Care

• Calling for help

• Assessing consciousness

• Pain assessment

Data is then presented to illustrate how decisions were made to inform these responses.

## Results

The midwifery students' response to the unfolding emergency in the scenario started with them:

### Rubbing up the fundus

The majority of students, 68% performed intermittent fundal massage. Only 30% of the students recognised the need to perform continual fundal massage. They did not allocate either themselves or the Dr in the room to perform this task, rather they tended to do this intermittently between other activities. Technique varied from incompetent to highly skilled. Variation in technique may have reflected fatigue notably among those who tried to perform continual massage. This finding indicates that this basic skill does require regular review to maintain currency and competent performance.

### Oxytocic administration-

Having recognised uterine atonia as the cause of post partum haemmorrhage, whilst rubbing up the uterus the student might be expected to call for assistance to draw up and administer uterotonic drugs (oxytocic drugs I/M) as well as order the citing of an I/V (Winikoff et al, 2010). In a midwifery emergency procedure this can be implemented without a medical order. In treating the cause of the bleeding immediately, there is the potential to prevent a small PPH from becoming a major PPH. However, the students demonstrated some considerable delay in enacting or requesting these interventions with approximately one third of them (13/35) not implementing any kind of management until more than 4 minutes had elapsed. At this stage the woman had already lost 1000 mls of blood and was beginning to show signs of shock.

Some students (18) focused on giving an oxytocic first and then moved to organising I/V access. Of these, fourteen requested Syntocinon and four requested Ergometrine or Syntometrine. Nine other students organized I/V access before administering an oxytocic (but not necessarily administering the oxytocic through the I/V line). Whilst eight other students simultaneously called for an I/V and oxytocic. If students were to do one thing first, it should be to administer the correct oxytocic.

### Blood loss

All the students checked blood loss. The students were stimulated to assess the pad after they knew the first BP reading was 95/70 and, on occasion, the actress alluded to feeling 'damp'. Laying the patient flat and administering additional oxygen were the most likely additional responses to those listed above.

### Bladder

Only 28% of the group considered inserting urinary catheters. The students had been trained to do this and were expected to be able to perform urinary catheter insertion in the immediate management of PPH.

### Calling for help

Ten students relied solely upon the Resident Medical Officer (RMO) in the room (i.e. not pressing the emergency buzzer or escalating calls for help to a MET or a CODE).

### MET

A medical emergency team was called to assist by 15 (43%) of the student midwives, 20 (57%) called for an emergency transfer to theatre by pressing the buzzer, and 3 (9%) made a resuscitation call (or CODE) with 13 (37%) students escalating calls for assistance by calling for help from more than one source. Of note, once back up from a medical emergency team or resuscitation crew has been initiated, the students were predisposed to slow their assessment and interventions.

Of the students who pressed the buzzer (n = 20), ten pressed within 4 minutes of the scenario commencing (range 0:47-3:25) with the rest calling after 4 minutes (range 4:10-6:44) when obvious clinical cues of deterioration had manifest.

### Loss of consciousness

The 'patient' started to lose consciousness from 4:30-6:00 minutes. Five students did not respond to the actresses 'faint/loss of consciousness' but continued with their actions in response to the blood loss and implementing the 4Ts algorithm. Fifteen (43%) students explicitly responded to the loss of consciousness by assessing whether Lisa (the mother) was alert, to voice, pain (by shaking) or unresponsive (AVPU). The loss of consciousness did stimulate the escalation for calls for help in all but three cases. Other responses included laying the patient flat, delivering additional oxygen, requesting bolus fluids and or second cannulation, and ordering further oxytocics.

### Pain assessment

Eighteen (51%) students assessed the mothers' pain, and of these, the majority noted pain in response to palpation/rubbing of the uterus. Only one of these students ordered pain relief for the mother (alongside fluids and oxytocics).

Algorithms have been developed to help students cope with emergency situations, however, where more than one procedure is recommended to be implemented simultaneously or within a very short space of time, the students struggled to prioritise their action. There were a number of significant issues that emerged from these data. They include the students:

1) remained calm throughout an extremely stressful experience (the use of actresses added to the sense of emergency and distress)

2) engaged with the woman and made an effort to explain what was happening

3) identified the problem although on occasions an accurate diagnosis was arrived at by default rather than through deliberation

4) drew upon a repertoire of behaviours that were relevant to that diagnosis, but not necessarily in a timely, logical order or related to emergent findings

5) appeared to only process one thing at a time. Some students focused on the woman's deterioration and implemented treatment for this, and once this was dealt with they moved onto the cause of the bleeding by giving oxytocics

6) The level of training (diploma, degree, masters), prior nursing experience seemed to have no significant effect on performance, although prior experience of emergency care and supportive clinical mentorship did.

### How were decisions formed?

The students had built a preliminary set of expectations before they entered the scenario. First, they had consented to participate in research examining deterioration. Secondly, the use of the simulation environment suggested an emergency scenario. The students were taught to be aware of four types of obstetric emergency. On entering the scenario they could immediately eliminate antenatal emergencies when they saw the baby in the cot and knew 'Lisa's' history (28 year old + primigravida + full term delivery). Therefore, before the students were conscious of honing in on the events of the scenario and what they meant, they had filtered out an enormous array of possibilities. Shortcutting to the cause of the emergency came from limited information that was assimilated from the initial experience of walking in the room:

'I thought she was going to have a PPH on the first one (scenario) because she had the baby' (Std 29, reflective interview).

'The lady was lying on the bed touching her baby - she seemed quite fine, talking - but with (the researchers) in the room .... I knew something was going to happen! This wasn't just a lady in the labour room - you know you had to be on the look out! (Student 15, reflective interview).

Whilst other students used these cues to hone in on possible emergencies that might unfold during the simulation.

'I thought - instantly .... because the baby was born it wasn't to do with birth-it could only be haemorrhage as the other obstetric emergency - so that was what I was thinking straight up - I was going through those scenarios thinking through those protocols'. (Std 1, reflective interview).

Here the students demonstrarte how they generate a hypothetical cause and then use clinical cues to confirm or refute their intial hunch of what was happening:

'the first thing was when she said she felt tired and her pulse was 110 my reaction was to look down below and feel her fundus and see how that felt' (Std 3, reflective interview).

'when I did the BP & realised it was low & felt the uterus & it was boggy that pretty much grounded my thoughts. OK, we are going down the PPH line' (Std 4, Reflective interview).

'It's always in the back of my mind a postpartum haemorrhage. I'm aware that can happen anytime ... so that is what I watching out for. ... I always go through, blood pressure, pulse and fundus. She said she felt cold so I went to check her fundus and check her loss and that's when I noticed a trickling that didn't stop.' (Std 25, reflective interview).

However, student 18 demonstrated an unsystematic assessment process that led to her discovering the PPH. Although she had made an initial decision about the emergency, she retrospectively gave importance to more subtle cues:

'When I walked in (Lisa) looked tired, but she had just had a baby, so there wasn't anything out of the ordinary. Perhaps she was guarding her stomach a little bit too much but not so sure. Attached to her baby. I only did some of her obs. I thought I might check her fundus and that's when I found it. She was bleeding.... I was expecting a PPH and then I went blank' (Std 18, reflective interview).

Student 7 articulated how the algorithm directed her cue seeking behaviour to build a clinical picture and account for the deterioration.

[Video footage - IV access requested and Std 7 feels Lisa's uterus] (she explains at reflective interview) - 'to see if it is contracting down. I am thinking tone, tissue, and thrombin. (laughs) it's in my head. We have complete placenta - so therefore I am thinking it can't be retention of products it has to be something else - it could be a clotting disorder - but I did tell the Dr what bloods to take (in the video footage the student did not make this request). That is a poor attempt at me trying to contract the uterus down by that manoevre .... [Video restarts - The student says: call a code] .... she is losing consciousness and the BP is really low - I didn't feel as though I needed to do any more observations as she was deteriorating and massaging the fundus to get some contraction going was the best I could do in that situation. Get the Dr to get the IV access and start giving some drugs and fluids'. (Std 7, reflective account on the video footage).

A cycle of deductive (working from the general possibility of a postnatal emergency and therefore what specific observations should be undertaken "top down") and inductive (building and collating specific observations and measures to assess patterns and trajectories to generate the clinical condition - "bottom - up") and back to deductive reasoning (testing the hypothesis of the diagnosis against specific cues) the students were able to capture what was happening in the scenario and decide how they should respond. Student 33 explains:

'I saw the baby in the cot .... You said about the BP, so I was already thinking about PPH ... she was feeling dizzy and light headed which is a definite sign of low BP and I was thinking bleeding. I went to feel the fundus and it was boggy so I wanted to rub that up so I could feel a contraction and I was thinking we might need some syntometrine. ....'

Of note the students tended to build single layers of information to inductively build diagnostic conclusions i.e. excessive postpartum PV blood loss = possible postpartum haemorrhage, the presence of a boggy uterus, indicative of retained products. The volume of PV blood loss stimulated them to ask for blood pressure and pulse readings - against a baseline they were able to detect the deterioration trajectory and thereby deduce hypovolaemic shock. The shock state was further reinforced when the mother lost consciousness along with abnormal oximetry recordings. Student 33 reflected on this

'I should've done CPR. I needed to bring her back. She was in trouble. That's when I wasn't sure what to do'. (The student had not assessed AVPU, nor assessed airway or circulation).

She did not call a CODE or the MET team.

Initially the student used the inductive-deductive cycle to test her hypothesis of PPH and hypovolaemic shock. However, when Lisa went unconscious Student 33 failed to inductively build a picture to support her hypothesis of cardiac arrest (or no volume output). She reflectively constructs the loss of consciousness to be due to no volume output, the trajectory of hypovolameic shock, without checking the mother's rousability, carotid pulse or respiratory pattern. In the reflective interview she fixates on this faulty diagnosis, but in the video she did not make a cardiac arrest call. This leaves one to suspect that she possibly added this hypothesis into the reflective review of the video to right herself perceived error rather than account for her decision-making in the scenario. This example demonstrates how the iteration between inductive and deductive reasoning helps to reduce the risk of fixation error but only when new data are introduced to refute or confirm the clinical decision (during the scenario or post hoc review of performance).

The response to single cues was evident in a number of the students:

'I was looking for trauma and then rubbing the fundus to involute ... I wanted to give her oxygen and lay her down. I put the oxygen on a bit slowly. ... I was asking for full bloods as she had lost a lot of blood. I was after volume expander. I did ask for O positive, but I thought we have to get her under control first so I asked for gelafusin. We could go with O positive when the results came back' (Std 3, reflective interview).

I was palpating her fundus to assess the tone, 'cos tone is one of the factors that could contribute to a PPH ...it felt little boggy so I rubbed it to encourage it to contract to help with the bleeding (5 mins 26 secs into scenario) ... she's about to go unconscious from excessive blood loss (speaking ahead of the footage), so I wanted to change her position to elevate her legs to encourage venous return to her heart to prevent any damage ... I ordered some large bore cannulas to be inserted in case she goes into hypovolaemic shock - so we can push fluids. I popped on some oxygen to maintain her airway because obviously that is the most important thing ... then I asked for 10 units of syntocinon to try get the uterus to contract because obviously my rubbing up wasn't effective (Std 12, reflective interview).

Here the student demonstrates how she rationalises her actions but attends to elements of the problem building incrementally to first respond to the volume depletion and then go back to address the possible cause. The error in her statement about oxygen delivery demonstrates how she recalls ABC but again does not apply a systematic response following the mnemonic to guide her emergency response. Further, her comment that oxygen delivery would protect an airway might indicate a shortcut in her processing a response to airway management and oxygen delivery.

A number of the students identified that working alone was problematic and impeded their response. The importance of other people was to act as a prompt to stimulate thinking as much as provide help in the response and share the responsibility for the clinical management of the mother.

'If it was a real case more people would have been there. ... You would have more than one brain to think things through. As it was an emergency situation it was difficult for me to concentrate and hold all the ideas in one. If there was another person there this reminds me to do this and this' (3 mins 18 seconds before the students implements any treatment) (Std 6, reflective interview).

'I reached a stage where I didn't know. I was hoping the MET team would come and take over. I don't know what to do next, hopefully someone comes and saves me (3 mins 22 seconds before the student implements treatment. Calls for a Dr at 3.41, calls the MET at 6.06) (Student 9, reflective interview).

'I wanted more people there,. I wanted to put synt into her leg and get some more in a drip later. I would have liked another pair of hands (4 mins 27 secs before treatment implemented, 7.07 before she calls the MET - not other back up was called) (Std 15, reflective interview).

I was a bit jumbled up. It was really scary. In reality I would not be the only midwife there (Buzzer 3.21, starts treatment 3.48 No other back up is called). (Std 16, reflective interview).

It is easier when you have got other people. ... but I can't rely on the fact that other people would be there as there could be another emergency going on. I think I would be OK but there were some things I would still be unsure about' (Buzzer pressed 4.52, starts treatment 5.17) (Std 32 reflective interview).

The students expressed how the simulation caused them to feel stressed and how they felt this anxiety affected their performance, in terms of how they made decisions and the order in which they responded. The presence of an external and preferably senior colleague was thought to enable them to pool their knowledge about what to do but also to provide a number of interventions that were required to stabilise the mother.

## Discussion

The students honed into the severity of the illness before they had entered the simulation. They had limited the possibilities of what might happen (obstetric emergency) and were automatically alert to give specific priority to certain stimuli [31]. This helped the students to control their cognitive processing albeit slow and serially [32] as they drew down from memory what to do in response to the clinical cues. Apart from the baby in the cot and the mothers' changing behaviour, all other information had to be sought. Building the data blocks to inform the diagnosis was deliberate and intense. However, the students, as novices, were slow in assimilating the clinical cues to determine the appropriate intervention. This was because they had to generate an idea of what was happening to focus their assessment to confirm or refute their initial hypothesis. All this had to be cognitively processed before reacting or making an emergency response. This mirrors Elstein and Bordage [33] three stage model of decision making:

• Hypothesis generation

• Cue interpretation

• Hypothesis evaluation

The delayed processing of clinical cues created 'bottlenecks'[34] to the students' emergency response. Their tendency was to right one problem then move on to right the next i.e. low blood pressure stimulated them to call for cannulation and then IV fluids; whilst loss of consciousness might stimulate them to lay the patient flat or elevate the legs, then administer oxygen and or then call for assistance. They were operating on individual rules to specific cues rather than a set of guided behaviours in response to a diagnosis (heuristic devices [35]). The dynamic deteriorating condition of the mother exacerbated the delay in attributing a single diagnostic label; the students were responding to the individual elements that built the deteriorating picture rather than responding to PPH. This might account for why they did not enact all of the requirements of clinical management using the 4 Ts sequentially. The mnemonic was helpful only if, and when, they had a chance to review what they had done after they had called for emergency backup.

The students used 'rules' to help them determine their response to certain clinical cues. They demonstrated a predisposition to act upon one cue rather than amass several cues (heuristic decision making) and respond to a diagnostic driven protocol to determine their response and in what order. This would sit with Benner's novice performance [35] and consistent with the students' limited clinical experience of midwifery emergencies. Even though the students had decided the scenario was about PPH in the very early stages of the scenario - this was often determined by factors other than the clinical presentation of the mother. The stress of the simulation meant the students were unable to systematically apply the guidance from a protocol. Rather they responded to individual triggers in the scenario e.g. low oxygen saturation the application of an oxygen mask, blood loss triggered a response to rub up the uterus. Other activities were initiated which implied a body of knowledge associated with PPH management e.g. ordering syntocinon but again under the stress of the scenario these 'rules' were not systematically and in some instances rationally applied.

A mnemonic is a useful strategy to help organize a response, but critically the student had to assimilate relevant cues and arrive at the correct decision to invoke the accurate protocol. Therefore, deeper assessment of how the midwifery students made decisions is important so we can hone these skills in training. Protocols cannot act as proxy to right performance if the initial decision on which to act is faulty or inaccurate [36]. Further, practice that is bound to one mnemonic could prohibit the students from invoking other protocols for example, early warning scores to signal the need for an emergency team response, rather than relying upon obstetric assistance (local doctor).

The students confirmed a dramatic PPH emergency was unfamiliar to them. The presence of an unhelpful Dr who failed to rescue or take command of the situation caused the students additional stress. In the reflective interviews the students complained of how unrealistic this was and contrary to their experience. The delay in arrival of the emergency response team to the students' calls for help exacerbated their anxiety. Of note, after the students had called for help their response to the dynamic deterioration of the woman slowed considerably. The students called for help once they considered their capacity to act had been exhausted not necessarily because they recognised a critical trajectory that required medical intervention in the future (however quickly that might arrive).

The dominant decision-making model of birth as a normal physiological and social activity may have affected this expectation. Risk, uncertainty and illness shifted the duty of care onto the obstetrician. The student midwives' first aid interventions to address hypovolaemic shock before the ERT arrived, were weak, poorly rehearsed and may well have represented the antithetical position to the dominant midwifery discourse (paradigm of care, the 'new midwifery' Porter et al add the reference number). Students were certain competent help would arrive and when it didn't it exacerbated their dilemma because the medical support in the room was so ineffective.

The majority of students did not instruct the Dr in the room to undertake tasks. They simply could not process multiple tasks and think ahead to instruct someone else because they were too focused on their own response. The Dr was particularly problematic because she did not think and act without prompting, nor was the Dr a source against which the student could check out and confirm her decision before acting. They had to think on behalf of the Dr and this contributed to confounding the complexity of the situation a feature the students said was unrealistic and to which they attributed, in part, their poor performance. Simulation provides an opportunity for students to address preconceived notions and expectations [36]. In this study, students had to confront the contradiction that they could not rely on others to lead an emergency response and they alone had to make decisions and provide an emergency response until help did arrive.

Cognitive processing and controlled searching for information (assessment) can be modified, learnt and reinforced [37]. The 'cognitive architecture' [38] of students can be sculpted to be more deliberate and precise in different types of situations, including working independently until help arrives. The benefit of learning team roles so the student can offer instruction to someone less experienced than themselves requires further research.

### Limitations

Students opted into the study. This self-selection process means that data should be viewed with some caution. The outcomes across the whole student cohort might illuminate greater variation in performance and decision-making than those reported from the 35 students who volunteered to participate. However, participants' biographical data indicated that the volunteers were fairly representative of their peers in age and range of experience.

In this study we were able to get students to articulate their thinking that informed action. The data were analysed to acknowledge different modes of thinking: analytical and responsive. The post review by students of their own video performance engaged them in reflective insight immediately after they had completed the scenario. This interview invited verbal retrospective, analytical insights that might have provided more conscious, deliberative and reason-based accounts than their initial performance indicated i.e. it appeared more intuitive, automatic, associative and fast, or in some cases, 'frozen' thinking.

Analysis of the reflective interview specifically addressed the sequencing of accounts relative to performance. It was noted that some students would speak ahead of their performance accounting for what they were about to do rather than what was happening in real time. Although this can be considered a significant limitation when considering the findings, other strategies such as speaking aloud decisions as they unfold would equally prompt analytical thinking rather responsive action more akin to live performance. In this design we were able to align this potential bias toward analytical thinking and balance that with the video vignette of actual performance and have provided these examples in the paper to illustrate this phenomenon.

### Fidelity vs replicability

The use of an actress served to heighten the students' engagement with the woman and her rapidly deteriorating health. The use of the birthing suit increased the fidelity of the simulation, enabled students to assess blood loss, assess the fundal height, and uterine contractility. What was gained in fidelity was potentially lost in terms of exact replication of performance in each scenario. The use of different patient actresses to play Lisa, and the interaction between the student and Lisa, meant there were some minor differences in each scenario (e.g. Lisa's position; the exact time at which a faint/loss of consciousness was simulated). On some occasions the actress prompted the student by offering information such as 'feeling damp', or by acting extreme agitation and expressing severe physical pain. However, the timelines for physiological changes were all standardized.

### Reflective Review

The use of reflective review to explore decision-making has great strength in enabling the students to gain insight into their own performance and limitations of knowledge. However, it is recognised that decisions made in action were recast when students reflected on their performance and gave an account of what they were thinking and doing at the time. Of note, the students were quick to remedy any serious defect (self evaluated) in their performance or give an account as to why they had made error (notably the contextual circumstances of the simulation, observers in the laboratory and the lack of fidelity to the real world).

Decisions that are viewed in hindsight are subject to cognitive bias and selective attention [29]. The facilitator did refocus the participant to explore specific aspects of their performance in the video if a student did not give an account of what was happening. This meant on occasion the researcher was deciding what was important, rather than allowing the student to determine what was meaningful and required explanation [22]. This happened on rare occasion, as the student did in the majority of cases stop the video where they felt they needed to explain or expand upon their performance.

In the reviews we noted that students would provide diagnostic labels ahead of their action in the video i.e. as their memory recall was prompted by the footage, they were able to determine the outcome of what they were currently (in the video) inductively building and assimilating data to inform a hypothetical proposition (possible diagnosis) that emerged late in the video footage. All the students left the simulation laboratory and went straight into the reflective interview without access to another student/colleague. Therefore, as they made this transition they were able to reflect upon the experience and make sense of the scenario. Prompted by the video recall they could superimpose their reflective decisions on the scenario ahead of the actions they made in the video. This is an important methodological issue for researchers seeking to make sense of student decision-making in action. The transcribed reflective interview needs to be aligned to action in the video to capture this reflective reconstruction. However, if the intention is to capture the participant's interpretation of events and their tacit understanding of their actions the researcher can gain valuable insight into the students' immediate recall, reasoning and feelings about the experience [22]. The immediacy of the review will impact upon the reflective reconstruction of events, but the researcher can still capture descriptions that help to draw schematic plans of the cognitive architecture in a simulated emergency response.

## Conclusions

Simulation laboratories provide an important safe environment for researching emergency decision-making and response skills. These data revealed that student midwives' clinical skills in response to an emergency require regular updating in the following areas

1) Fundal massage technique;

2) the use of standing order drugs to address haemorrhage;

3) when to call for help and escalate calls for emergency assistance and review what type of team is required under certain circumstances;

4) the use of IV fluids to replace volume depletion;

5) prioritizing interventions from an algorithm when working independently;

6) implementing independent emergency response until help arrives;

7) delegating tasks to others in an emergency; exposure to structured reflection. Although not always possible in the clinical situation,

8) opportunities for de-brief with a mentor should become embedded into practice and actively sort by the student.

Students would benefit from learning expressly about how they make decisions in crisis and how they can use heuristic devices to make diagnoses and mnemonics to evaluate their interventions. Notably students need to become aware of how they tend to operate in response to individual rules relating to specific cues rather than a set of guided behaviours in response to a diagnosis. The regular checking back between inductive and deductive reasoning can reduce fixation error and risk of a faulty action.

## Abbreviations

CEMACH: The Confidential Enquiry into Maternal and Child Health; ERT: Emergency Response Team; DA: Dimensional Analysis; MET: Medical Emergency Team; PPH: Post Partum Haemorrhage; The 4 Ts: (Tone Tissue: Trauma: Thrombin).

## Competing interests

The authors declare that they have no competing interests.

## Authors' contributions

JS, RE, MA-B, BB, SC, LK, were responsible for the study conception and design. SC, BB, MA-B, JJ, MM, CG, RB, LK, collected the data and JS. MA-B. BB FZ performed the data analysis. JS drafted the manuscript and BB, MA-B, RE and SC made critical revisions to the paper for important intellectual content. All authors read and approved the final manuscript.

## Pre-publication history

The pre-publication history for this paper can be accessed here:

http://www.biomedcentral.com/1471-2393/12/19/prepub
